# Preparation and Self-Cleaning Performance of Carbon-Based Superhydrophobic Coatings Based on Non-Fluorine and Non-Toxic Corn Straw

**DOI:** 10.3390/molecules26216401

**Published:** 2021-10-22

**Authors:** Yanbin Wang, Lihui Kang, Zhaoxia Li, Qiong Su, Shaofeng Pang, Lichun Liang, Dian Wang, Shijun Cao

**Affiliations:** 1 School of Chemical Engineering, Northwest Minzu University, Lanzhou 730030, China; ybwang@126.com (Y.W.); lihuikang0325@163.com (L.K.); pangshaofeng2006@163.com (S.P.); fgl770880llcfgl@163.com (L.L.); d_wang1997@163.com (D.W.); a2848695227@163.com (S.C.); 2 Key Laboratory of Environment-Friendly Composite Materials of the State Ethnic Affairs Commission, Lanzhou 730030, China; 3 Key Laboratory of Utility of Environmental Friendly Composite Materials and Biomass in Universities of Gansu Province, Lanzhou 730030, China; 4 Engineering Research Center of Biomass-Functional Composite Materials of Gansu Province, Lanzhou 730030, China

**Keywords:** superhydrophobic, self-cleaning, eco-friendly, dip-coating, pyrolysis

## Abstract

Recently, superhydrophobic surfaces with self-cleaning ability have attracted broad research interest due to their huge potential in daily lives and industrial applications, but the use of fluorinate, toxic organic compounds, and expensive feedstocks make superhydrophobic materials a great challenge in practical application. In this study, we present a facile dip-coating strategy to prepare superhydrophobic coatings with self-cleaning properties based on a non-fluorine and non-toxic system by using eco-friendly corn straw as raw material. During this process, aromatic carbon particles with rough hierarchical structures were prepared firstly via a simple fast pyrolysis process, followed by modification with polydimethylsiloxane (PDMS) in absolute ethanol solvent to decrease the surface free energy. Research shows these natural straw-derived carbons display a microstructure of several protrusions which is similar to the lotus leave’s and the resulted coatings exhibit an outstanding superhydrophobic property with a static water contact angle (WCA) of 151.67 ± 1.36 degrees. In addition, the as-prepared coatings possessed excellent self-cleaning performance: no contaminations were observed on the surfaces after examining with sludge, calcimine, water, and common liquids such as tea, milk, soybean milk as well as ink, which have a broad range of potential application in the field of antifouling, waterproofing, and anticorrosive.

## 1. Introduction

In recent years, with environmental pollution intensifies, there are increasingly suspended particles and dust in the air, which leads to the increasing possibility for the surfaces exposed to the ambient atmosphere such as ships, automobile windshields, external wall and glass of high-rise buildings, windmill’s sails, etc. to be contaminated, which results in not only increasing cleaning cost but the difficulty to clean [1,2,3,4]. Therefore, it is very imperative to explore a contamination-free surface to improve dirt tolerance.

Superhydrophobic surfaces with self-cleaning properties have been proposed as a terrific way to turn contamination-free surfaces into reality. Superhydrophobic materials surfaces are defined as a surface that displays a water contact angle greater than 150° along with roll-off angles less than 10° [5]. The performance of superhydrophobic surfaces is covered by two critical parameters: low surface energy and rough micro-/nanostructures. Correspondingly, there are two main routes to fabricate superhydrophobic surfaces at present: (a) creating rough micro-/nanostructures on hydrophobic surfaces (water contact angle larger than 90 degrees), and (b) chemical modification of a micro-/nanostructured substrate with hydrophobic modifier [6,7]. For the realization of low surface energy, a large number of chemicals have been reported, such as fluorine-containing compounds, long-chain organosilanes, and fatty acids. With tremendous efforts devoted to the superwettability mechanism of organisms in nature including water striders legs [8], butterfly wings [9], rose and sunflower petals [10], lotus plant leaves [11], gecko foot [12], etc., a wide range of methods and techniques have been developed to texture hierarchical micro-/nanosized structures which can be divided into two design strategies: one is fabricated from larger-scale materials and the another is manufactured from atomic or molecular scale materials [13]. In more detail, these methods and techniques involve lithography [14], diamond cutting [15], etching [16], 3D printing [17], layer-by-layer assembly [18], self-assembly [19], sol-gel [20], and others.

Physical nanoparticles are an effective strategy to provide hierarchical micro-/nanostructure. Currently, various kinds of synthesized nanoparticles are used in superhydrophobic performance. For instance, Zhong et al. reported a self-cleaning superhydrophobic surface with excellent self-cleaning properties and water repellency by extracting lignocellulose nanofibrils from wheat straw followed by modification of 1H, 1H, 2H, 2H-perfluorooctyltriethoxysilane [21]. Chen et al. fabricated self-cleaning and superhydrophobic surfaces on various substrates through coating with calcium carbonate nanoparticles modified with perfluorooctyltriethoxysilane [22]. Additionally, Nine et al. prepared graphene-based superhydrophobic coatings with favorable self-cleaning and anti-corrosion ability via mixing diatomaceous earth, synthesized graphene oxide, and TiO_2_ in THF solution followed by modifying with PDMS [23]. Lv et al. created a robust and nonfluorinated superhydrophobic coating by using silicon resin as a low surface energy modifier and reinforced fillers, carbon nanotubes/graphene as a rough structure which displayed excellent self-cleaning and anti-wear performance [24]. However, it could be concluded from the mentioned works that raw materials and the processes used for conferring superhydrophobicity to natural polymer involve some shortcomings, such as, high feedstock costs, complicated preparation process, time-consuming reaction, fluorinated compounds, and toxic organic solvents, causing negative effects to human health and natural environments [25,26]. Thus, the fabrication of new environmentally friendly and biodegradable superhydrophobic coatings is extremely crucial to the sustainable development of humanity and nature.

As a kind of widely distributed, low-cost, biodegradable, and environmentally friendly resource, biomass materials such as straws, falls, trees, etc., have attracted extensive research interests in an effort to fabricate superhydrophobic materials. Carbon nanoparticle is a carbonaceous material that can be easily fabricated through the pyrolysis process of biomass waste. Therefore, in this article, we present a straightforward dip-coating method to prepare superhydrophobic coatings on different substrates based on easily generated carbon particles by utilizing low-cost materials and non-fluorinated chemicals without any toxic reagents and expensive equipment, exhibiting great potential for large-scale production. The obtained coatings not only displayed high water repelling properties but also showed excellent self-cleaning performance. Above all, this work provides an economical, facile, and eco-friendly approach to prepare superhydrophobic self-cleaning surfaces which have great potential application in water collection, antifouling, oil-water separation, anticorrosion, and anti-icing. On the other hand, for the resource shortage reason, the present strategy provides a novel insight into the further utilization of biomass waste.

## 2. Results and Discussion

**Figure 1:**
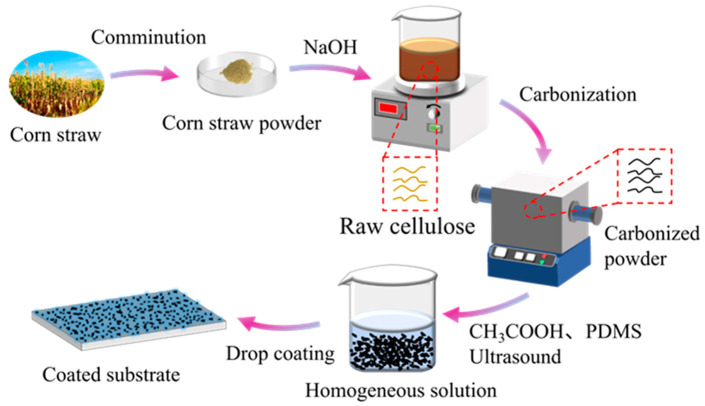
Schematic diagram of the preparation procedure for the superhydrophobic coatings.

The preparation process of superhydrophobic coatings is illustrated in Figure 1. This strategy possesses a simple two-step efficient process. First, amorphous carbon nanoparticles were prepared via fast pyrolysis of corn straw at different temperatures to form a rough structure. Subsequently, superhydrophobic surfaces were fabricated by dip-coating the ethanol-based suspension of carbon particles/PDMS composites onto various substrates.

**Figure 2:**
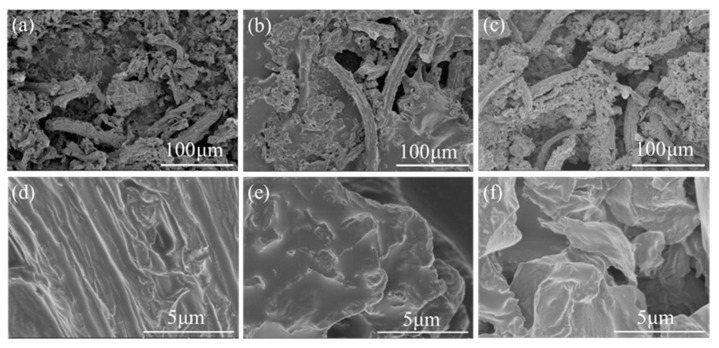
SEM images of CPs at carbonization temperature of (**a**,**d**) 400 ℃; (**b**,**e**) 600 ℃; (**c**,**f**) 800 ℃. (**d**–**f**) are enlarged images of (**a**–**c**), respectively.

It is well known that the wettability of surfaces is governed by the chemical composition and micro/nanostructure [27]. To further understand the superhydrophobicity mechanism of the as-prepared samples, a scanning electron microscope (SEM) was used to characterize the surface microstructure of CPs carbonized at different temperatures, as shown in Figure 2. Clearly, the micromorphology of CPs is shown as rod-like structures with several protrusions. It can be concluded that the specific area and the roughness of CPs samples are noticeably enlarged. As Figure 2a–c shows, with increasing pyrolysis temperature, CPs display an increase in the number of rod-like structures. Furthermore, it can be seen that there is a larger number of punctiform protrusion in Figure 2e which is a structure similar to the lotus leaf [28] on the surface of CPs-600 compared to CPs pyrolyzed at 400 ℃ and 800 ℃.

**Figure 3:**
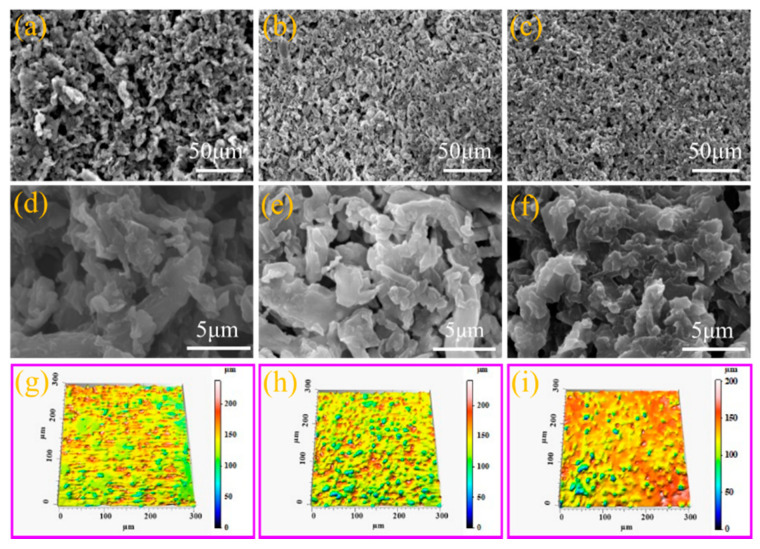
The surface morphologies (**a**–**f**) and 3D contour images (**g**–**i**) of the resulting coatings. (**a**,**d**,**g**) PDMS/CPs-400; (**b**,**e**,**h**) PDMS/CPs-600; (**c**,**f**,**i**) PDMS/CPs-800.

Figure 3 displays the surface morphologies and 3D contour images of PDMS/CPs-400, PDMS/CPs-600, and PDMS/CPs-800 coatings. As exhibited in Figure 3a–f, porous coatings were formed after curing owing to the stacking arrangement of nanoparticles. It can be seen from Figure 3a–c that there are more porous coatings on the surface of PDMS/CPs-400 coating, which might be caused by the incomplete pyrolysis process at the lower temperature of 400 °C. Compared with SEM images, the surface roughness can be seen more intuitively from the 3D contour images. It is obvious from Figure 3g–i that non-uniform protrusions are visible on the surface of PDMS/CPs coatings. For PDMS/CPs-400 coating, there are almost no apparent protrusions observed, indicating a relatively smooth surface morphology. The morphologies of the protrusive microstructure are improved on the surface of the PDMS/CPs coating with the treatment temperature increased to 600 °C. Figure 3i clearly shows that there are more red areas and the blue area is almost invisible. Simultaneously, fewer protrusions can be observed on the surface of the PDMS/CPs-800 coating. This suggests that a flatter surface was formed under the pyrosis temperature of 800 °C. The roughness average (Ra), a most frequently used surface topography parameter, was employed to probe the impact of pyrosis temperature and the results of measurements for the resulting coatings are shown in Table 1. In Table 1, the roughness values, the mean values, as well as the standard deviations are given for each coating. It is found that the Ra mean value of PDMS/CPs-400 coating is 2.87 μm. With the pyrosis temperature increased to 600 °C, Ra reached the largest value of 3.34 μm. However, when the pyrosis temperature increased to 800 °C, the value of Ra decreased to 2.29 μm. The results of the surface morphologies analysis reveal that as pyrolysis temperature increased, the surface roughness exhibit first an increasing trend and then decline, which corresponds to the analysis results of the CPs microstructure. It can be concluded that the changes of surface morphologies consistent with the microstructure of CPs and the PDMS/CPs-600 coating could better prevent the water from contacting the substrate.

**Figure 4:**
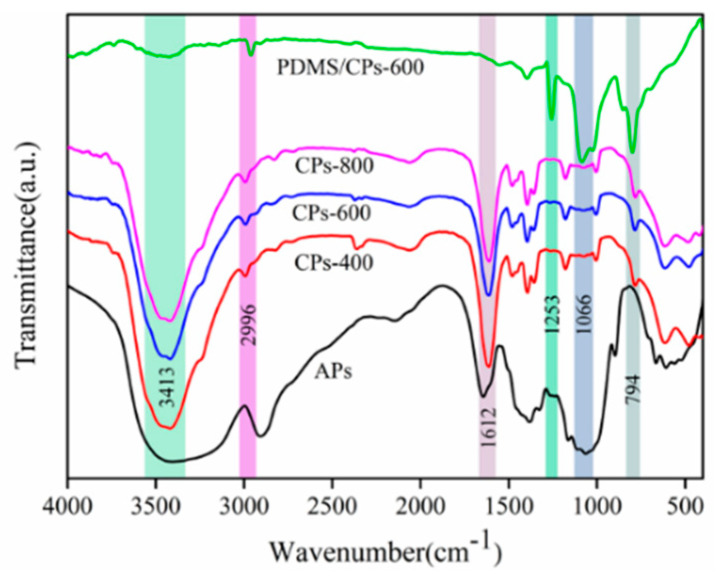
FT-IR spectra of APs, CPs-400, CPs-600, CPs-800 and PDMS/CPs-600.

To investigate the evolution of the chemical structure of raw corn straw fibers, carbonized particles, and coatings modified with PDMS, the FT-IR spectroscopy was taken over the wavenumber range of 400 to 4000 cm^−1^ and the results are shown in Figure 4. As indicated, the surface components were changed after carbonization and modification. Besides, it is obvious to find that the IR spectra of straw carbon-based materials fabricated at different temperatures show similar absorption peak features, suggesting the biochars have already formed at the low temperature of 400 °C. The broad band at approximately 3413 cm^−1^ corresponds to the O–H stretching vibrations of the carboxyl and phenolic hydroxyl groups—it is consistent with the literature report [29]. However, the peak intensity of the PDMS/CPs-600 product at around 3413 cm^−1^ was weak which indicating a decrease in the number of O−H groups and the formation of hydrophobicity. The adsorption peaks appearing at 2996 cm^−1^ and 2908 cm^−1^ belong to the stretch vibration of the -CH_3_ group. The sharp adsorption band around 1612 cm^−1^ is vibration from C=O and C=C stretching, implying the existence of an aromatic group [30]. Moreover, the adsorption peak of the raw straw at 1066 cm^−1^ assigned to the C–O group disappeared after pyrolysis which indicates that the crystallized region of the corn fibers was disrupted and the polysaccharides in the straw had completely degraded at the temperature of 400 °C [31]. In addition, the FT-IR spectra of modified carbon particles (PDMS/CPs-600) show the new adsorption peaks near 1253 cm^−1^, 1066 cm^−1^, and 794 cm^−1^, which are assigned to the C−H group in Si−CH_3_, Si−O−Si asymmetric, and symmetric stretching vibration [32,33], implying that PDMS was successfully grafted onto the CPs.

**Figure 5:**
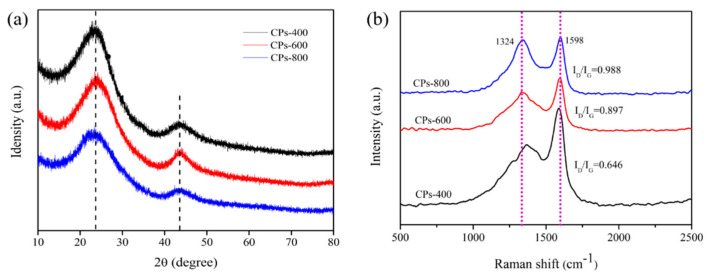
XRD pattern (**a**) and Raman spectra (**b**) of CPs-400, CPs-600, and CPs-800.

As we can see from the result of the SEM analysis, the structure of straw carbon particles formed at different temperatures differs from each other. To understand explicitly these changes, XRD analysis was employed to study the crystal structures of CPs pyrolyzed at different temperatures. As depicted in Figure 5a, two characteristic broad peaks at 2θ = 23.7° and 2θ = 43.4° occur in all cases which correspond to the (002) plane and the (100) plane of disordered graphitized carbon, respectively [34]. The peak exhibits at the low angle of 23.7° suggesting the existence of a large number of amorphous carbon particles [35] which might be due to the direct carbonization without any other chemical treatment and this is in accordance with published work. In addition, the formation of amorphous carbon in the process of pyrolysis indicates damage to the cellulose crystal structure. Moreover, the presence of a diffraction peak at around 23.7° (002) of each sample verifies the presence of different micropore-wall structural units [36]. A close examination of the diagram shows that the peaks near 23.7° shift slightly to the lower angle with the increasing pyrolysis temperature, indicating that the interplanar spacing of CPs increases. As concluded from these results, the change of pyrolysis temperature has a significant influence on the crystal structure of CPs.

For further structural characterization, CPs-400, CPs-600, and CPs-800 were characterized by a Raman microspectrometer. As exhibited in Figure 5b, there are two relative peaks of CPs at around 1324 cm^−1^ and 1598 cm^−1^ corresponding to the D bond of amorphous carbon and G bond of Graphite carbon, respectively [37,38,39]. It is found that the intensity ratios (I_D_/I_G_) for CPs-400, CPs-600, and CPs-800 are 0.646, 0.897, and 0.988 which shows a low degree of graphitization. It also can be seen that the value of I_D_/I_G_ increased gradually with increasing heat treatment temperature. This was mainly caused by the increasing surface defects of CPs. Meanwhile, the CPs-800 sample possessed the highest I_D_/I_G_ value against other samples which indicates a higher extent of disorder in the CPs [40].

**Figure 6:**
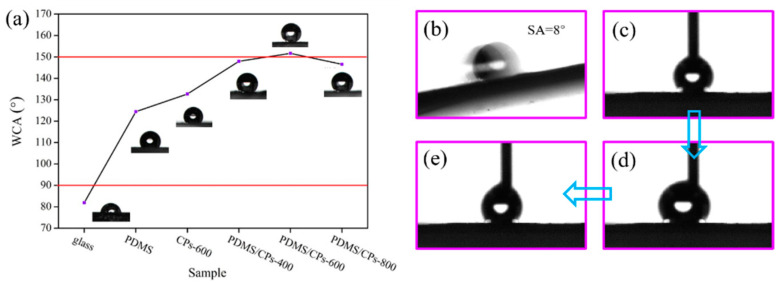
(**a**) The static water CAs of glass, PDMS coating, 600 ℃ CPs and PDMS/CPs glass substrate with carbonization temperature of 400 ℃, 600 °C, and 800 ℃; (**b**) The SA test of PDMS/CPs-600; (**c**–**e**) Time sequence pictures for hysteresis angle of PDMS/CPs-600.

The water contact angle (WCA) test is important to determine the wettability of the as-prepared coatings. The results are shown in Figure 6a. During this process, the glass samples prepared from different coating solutions were taken as the test object. As can be seen in Figure 6a, the static water contact angles vary from 146.56 ± 2.14° to 151.67 ± 1.36° and the PDMS/CPs-600 coating exhibited the biggest contact angle value of 151.67 ± 1.36°, indicating its outstanding superhydrophobicity. Moreover, the WCA of PDMS/CPs-400 and PDMS/CPs-800 coatings were measured to be 147.96 ± 1.36° and 146.56 ± 2.14°, respectively, which shows good hydrophobicity of those two surfaces. All these results demonstrated that the performance of non-wettability increased with an increase in carbonization temperature, and the carbonization temperature of 600 °C formed the best hydrophobic property. In order to better understand the superhydrophobic performance of PDMS/CPs-600, the measurements of sliding angle (SA) and contact angle hysteresis were carried out at ambient temperature with a 2 μL water droplet, as shown in Figure 6b–e. The obtained SA value is 8° lower than 10°and hysteresis angle is 4.1 ± 2°, indicating an outstanding superhydrophobicity of PDMS/CPs-600 coating.

**Figure 7:**
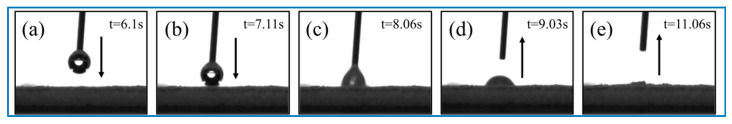
Time sequence photographs at (**a**) t = 6.1 s, (**b**) t = 7.11 s, (**c**) t = 8.06 s, (**d**) t = 9.03 s and (**e**) t = 11.06 s for wettability measurement of the raw material with a 2 μL water droplet, the arrows stand for the needle moving direction.

The wettability behavior of the original glass slide, glass coated with PDMS, and CPs-600 coated onto a glass surface were also evaluated. As shown in Figure 6a, the original glass exhibited hydrophilic with a WCA value lower than 90 [27]. After introduction of PDMS, the hydrophilic original glass came to be hydrophobic (WCA = 124.44 ± 1.26°), indicating the excellent inherent hydrophobicity of PDMS. The water contact angle of PDMS/CPs-600 is shapely higher than PDMS and CPs-600, which suggests that PDMS was successfully grafted onto the surface of CPs. This result is consistent with the FT-IR analysis. Meanwhile, as seen in Figure 7, the dropped water was absorbed immediately by raw straw fibers, indicating hydrophilicity of raw material, which is due to the existence of numerous hydrophilic groups. By contrast, the water CA of CPs without modification is approximately 132°, which might be due to the decline in the number of polar functional groups of corn straw after pyrolysis at high temperature, as shown in the infrared spectrum. A very important result that can be drawn from the above discussion and SEM analysis is that the micro/nanostructure is one of the key factors of superhydrophobic performance.

**Figure 8:**
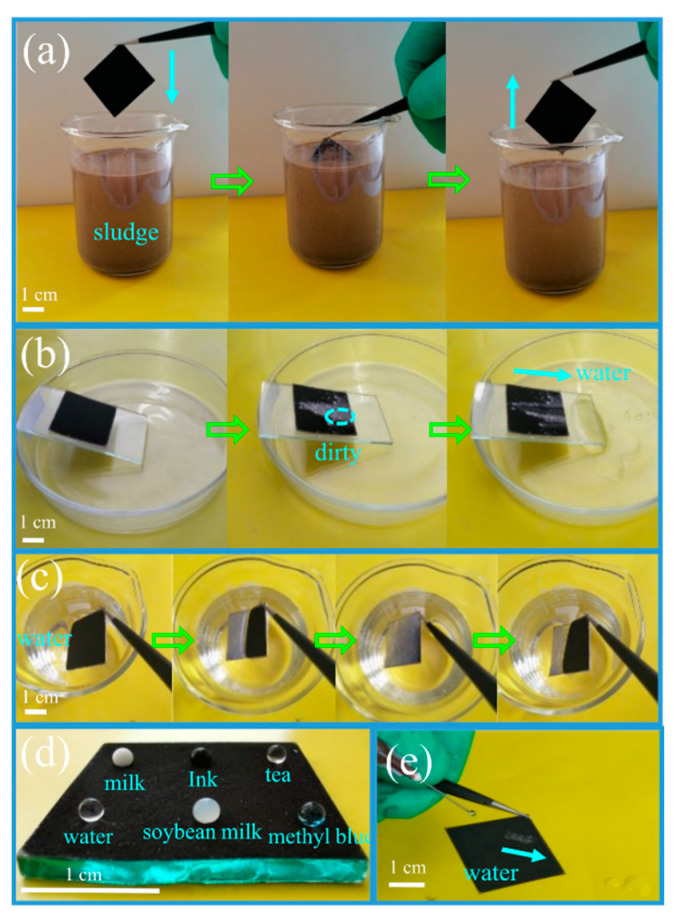
Photos of self-cleaning tests of the as-prepared coating. (**a**) antifouling test process; (**b**) time sequence of white wall ash carried away by water; (**c**) the silver mirror phenomenon of the sample under water; (**d**) photographs of water and various representative stains on the coated substrate; (**e**) image of water jet attacking the coating.

In order to demonstrate the self-cleaning properties of the superhydrophobic coatings, various self-cleaning tests were carried out on the surface of PDMS/CPs-600 under room temperature. In these experiments, sludge and white wall ash were treated as contaminants, meanwhile, the milk, ink, tea, and soybean milk were used as common household liquids. When the as-prepared coating material was taken out after soaking in the sludge, as expected, there were no visible contaminants on the superhydrophobic surface (Figure 8a, Supplementary Video S1), which might be caused by the small adhesion force of dirt on the coated surface [41]. However, the uncoated sample was contaminated obviously during a similar process—shown in Supplementary Video S1. Coated and uncoated substrates comparison showed an excellent antifouling property of the as-prepared coating. As shown in Figure 8b, the water was dropped on the superhydrophobic surface that was contaminated by white wall ash and the droplets rolled off rapidly with the dust without wetting the coated slide, illustrating an excellent dirt-removal property of the as-prepared coatings. Figure 8c shows that a silver mirror phenomenon on the superhydrophobic coating surface was observed when it was immersed in water, and then it floated on the surface of the water once it lost the external force, furthermore, the coated slide retained its non-wetting property after being moved out from the water. As shown in Figure 8d, different liquid droplets, simulated as real dirt conditions, are spherical in shape on the surface of the superhydrophobic coating, thus, the modified surfaces possess excellent antifouling performance. Besides that, Figure 8e shows an optical photograph of a jet of water bounced off and slipped off without any water spread out on the coated surface (also shown in Supplementary Video S2), conversely, the water droplets remain on the uncoated substrate and leave the surface in the form of water flow (Supplementary Video S2), indicating its outstanding waterproof ability. All these experimental results reveal that the corn straw carbon-based superhydrophobic surfaces modified with PDMS have great potential application in the fields of self-cleaning, anti-fouling, anti-corrosion, water collection, and anti-icing.

**Figure 9:**
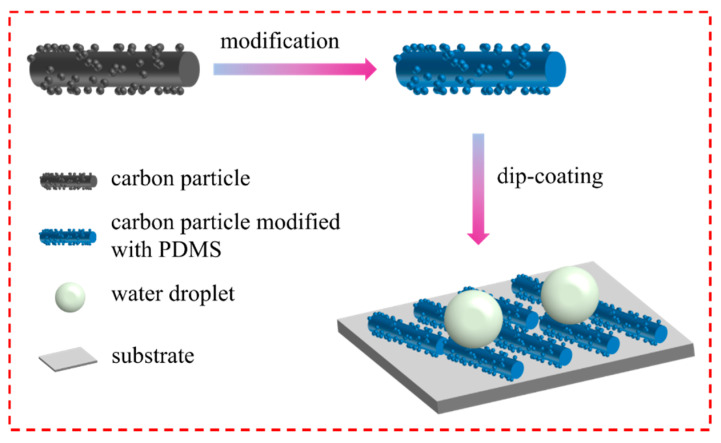
Schematic diagram for the superhydrophobic mechanism of PDMS/CPs coating.

On the basis of these results, the superhydrophobic mechanism of the as-prepared coatings with self-cleaning ability was proposed (Figure 9). The microstructure of CPs pyrolyzed at the temperature of 600 °C is a kind of rod-like structure with several protrusions. Moreover, the results of the WCA measurement showed that superhydrophobic surfaces fabricated by CPs pyrolyzed at 600 °C have the highest value. Therefore, we can conclude that the structure’s material plays an important role in hydrophobic performance. Besides, the PDMS used in the superhydrophobic coatings preparation procedure is a hydrophobic polymer with advantages of outstanding chemical stability, transparency flexibility bio-compatible [42,43,44], etc. When PDMS was mixed with CPs in an ethanol solution, the crosslinked PDMS film formed on the surface of carbon particles which effectively decreased the surface energy of PDMS/CPs coatings [45].

## 3. Materials and Methods

### 3.1. Materials and Chemicals

Corn straw was collected from a farm near the Yuzhong campus of Northwest Minzu University (Lanzhou, China); glass plates (25 mm × 25 mm), titanium plates (25 mm × 25 mm), and aluminum plates (25 mm × 25 mm) were supplied by Zhongnuo Advanced Material (Beijing, China) Technology Co., Ltd.; PDMS, Sylgard-184 with components of PDMS base and curing agent purchased from Dow Corning Company; sodium hydroxide was provided by Shuang Shuang Chemical Co. Ltd. (Yantai, China). All chemicals were used as received without further purification and deionized water (DI water) was used throughout all experiments.

### 3.2. Pre-Treatment of the Corn Straw

After air drying, the corn straw was cut into approximately 3 cm lengths followed by thoroughly cleaning with water and then placed in a thermostatic blast drying oven and dried for 12 h at 80 °C. Following this treatment, corn straw was ground by a high-speed pulverizer to obtain straw powders, and then the powders were passed through a 100-mesh sieve to collect uniformly graded powders. Afterwards, 20 g of corn straw powders was added in 400 mL of 5 M sodium hydroxide solution and the mixture was kept at room temperature under magnetic stirring for 2 h. Next, 600 mL of deionized water was added into the viscous solution and the straw fibers were collected by filtration, followed by adjusting the pH to 5–6 with H_2_SO_4_ (1 M). After washing with distilled water several times, the pretreated corn straw was dried at 80 °C overnight. Then, the alkalized straw powders (APs) were obtained.

### 3.3. Preparation of Corn Straw Based Carbon Particles (CPs)

The straw carbon particles were fabricated by the thermal pyrolysis method. In detail, 4 g CPs was placed into a tube furnace and heated from room temperature to 400 °C under a nitrogen atmosphere with a heating rate of 5 °C/min and kept for 1 h. Thereafter, the temperature decreased naturally to the ambient temperature. The produced carbon particles were denoted as CPs-400. In order to probe the effect of pyrolysis temperature on the superhydrophobic performance of PDMS/CPs coatings, CPs-600 and CPs-800 were prepared under the carbonization temperature of 600 and 800 °C according to the same procedure.

### 3.4. Fabrication of Coating Suspension

The first step in this process was to transfer 0.1 g (1.8 wt%) of above as-prepared CPs to a mixture solution containing 7 mL absolute ethanol and 0.05 g of the PDMS precursor. The mixture was sonicated for 30 min to form a uniform dispersion. Subsequently, a curing agent was added (base to curing agent at a weight ratio of 10:1) and then the mixture was ultrasonically dispersed for another 15 min for the formation of coating suspension.

### 3.5. Fabrication of Superhydrophobic Coatings

The superhydrophobic coatings were prepared via a simple drop-coating process. Before use, a glass sheet, aluminum sheet, and titanium sheet were thoroughly washed with ethanol and distilled water, then dried in the oven at 80 °C for 30 min. Then, 0.5 mL coating suspension was drop-coated onto a glass sheet, aluminum sheet, and titanium sheet by a dropper at a speed of one drop per second. Finally, the coated substrates were cooled down naturally at room temperature, followed by drying in a thermostatic blast drying oven at 80 °C for 30 min to allow the full curing of PDMS and complete ethanol evaporation. This preparation process was repeated once more and the resulting coating thickness was measured to be 75 ± 5 μm. The fabricated superhydrophobic coatings were designated as PDMS/CPs-400, PDMS/CPs-600, and PDMS/CPs-800.

### 3.6. Characterization

The surface morphologies of CPs in the pyrolysis process at different temperatures were studied by scanning electron microscopy (SEM, SU8000, Hitachi High-Technologies Corp.; FESEM, JSM-6701F, Japan Electronics corporation, Tokyo, Japan). Fourier transformed-infrared spectroscopy (FT-IR, Nicolet 380, Thermo Electron Corporation, Waltham, MA, USA) was employed to analyze the change of components and structure of APs, CPs, and PDMS/CPs-600 in a scanning range from 400 to 4000 cm^−^^1^. X-ray diffraction (XRD) was recorded on a diffractometer (X’Pert PRO, PANalytical, Almelo, Holland) at 40 kV and 40 mA in the scanning range of 2θ = 10−80° with a speed of 10°/minute. Raman spectra were obtained in the 500–4000 cm^−1^ range with 532 nm laser excitation using a Raman spectrometer (Dxr 2Xi, Thermo Fisher Scientific, Waltham, MA, USA). The wettability of the raw straw particles, CPs-600, PDMS, and substrate surfaces coated with and without PDMS/CPs was measured by static water contact angle at ambient temperature through a contact angle goniometer (HARKE-SPCAX3, Beijing, China). During this procedure, a volume of 2 μL deionized water droplets was used for each measurement and the reported value of each sample was an average of five different positions. The resulting surface roughness was measured by a 3D optical profilometer (MicroXAM-800, KLA-Tencor Corp., Milpitas, CA, USA).

## 4. Conclusions

In this work, biochar-based fluorine-free and non-toxic superhydrophobic coatings with self-cleaning ability were fabricated on various substrates via facile pyrolysis carbonization and dip-coating strategies from low-cost, renewable, and widely distributed corn straw. The reported superhydrophobic coatings exhibited a high static water contact angle of 151° in air and excellent anti-fouling and nonwetting performance in self-cleaning experiments. In addition, we studied the effect of pyrolysis temperature on the superhydrophobic performance of PDMS/CPs coatings and the temperature of 600 °C is determined to be the optimal value. In summary, the facile, low-cost, and environmentally friendly fabrication strategy of the PDMS/CPs coatings with an outstanding self-cleaning property is expected to promote the efficient utilization of agricultural resources and have great potential applications in the self-cleaning field.

## Figures and Tables

**Table 1:** Surface roughness parameters for the resulting coatings of PDMS/CPs-400, PDMS/CPs-600 and PDMS/CPs-800.

Sample	Ra (μm)			Mean Value (μm)	Standard Deviation (μm)
	1	2	3		
PDMS/CPs-400	2.74	2.83	3.05	2.87	0.13
PDMS/CPs-600	3.35	3.14	3.52	3.34	0.16
PDMS/CPs-800	1.83	2.68	2.36	2.29	0.35

## Data Availability

The data presented in this study are available on request from the corresponding author.

## Supplementary Materials

The following are available online. Video S1: Antifouling measurements of coated and uncoated aluminum plates; Video S2: Water jet tests of coated and uncoated aluminum plates.

## Sample Availability

Samples of the compounds are not available from the authors.
